# DNA barcoding and geometric morphometry of tabanid flies (Diptera: Tabanidae) in Thailand and a new record of a Thai horse fly

**DOI:** 10.1111/mve.70058

**Published:** 2026-02-27

**Authors:** Nantatchaporn Klaiklueng, Weluga Bootsongkorn, Patsharaporn T. Sarasombath, Pichet Ruenchit, Sirichit Wongkamchai

**Affiliations:** ^1^ Department of Parasitology, Faculty of Medicine Siriraj Hospital Mahidol University Bangkok Thailand; ^2^ Siriraj Integrative Center for Neglected Parasitic Diseases, Department of Parasitology, Faculty of Medicine Siriraj Hospital Mahidol University Bangkok Thailand

**Keywords:** checklist, *Chrysops*, geometric morphometrics, Narathiwat, Phayao, Southeast Asia, species delimitation, *Tabanus*, vector surveillance

## Abstract

Tabanid flies are gaining high medical and veterinary importance due to their role as a vector of many pathogens. In the present study, a total of 3760 female tabanid flies were collected from Narathiwat and Phayao provinces of Thailand. All were identified using the morphological method, DNA barcoding and wing geometric morphometric (WGM) analysis. Eight species were identified, and among them, *Tabanus tenens* is a new recorded Thai horse fly. Morphologically, 2178 and 1559 females from Narathiwat and Phayao were identified at the species level, including *Chrysops dispar*, *Chrysops fasciatus*, *Tabanus griseilineis*, *Tabanus rufiscutellatus* and *Tabanus minimus*. The other 23 females were identified at the level of the genus (*Tabanus* spp.) only. Among these, DNA barcoding was further identified as *Tabanus tenens*, *Tabanus rubidus* and *Tabanus striatus*. The landmark‐based WGM analysis was used to differentiate the samples from Narathiwat, and the results showed the efficacy of this approach in differentiating the four species of tabanids, achieving an overall accuracy score of 99%. Additionally, the data derived from wing landmarks of samples collected in Narathiwat were used as reference materials for identification of the tabanid fly collected from Phayao, and the finding revealed efficacy of the reference materials. Together, this study demonstrated that DNA barcoding is a reliable tool for the identification of tabanid fly species, while WGM analysis could be a complementary tool. The barcode sequences and WGM data generated in this study can serve as a valuable reference material to identify new field samples from other regions of Thailand. Altogether, this study updated the species list of tabanid flies in Thailand, particularly in the Narathiwat and Phayao provinces, using various integrative identification tools.

## Graphical abstract

- *Tabanus tenens* identified in this study is a newly recorded species in Thailand.
- DNA barcoding using the *cox*1 gene identified tabanid species in the present study.
- WGM data from different sites served as a reference for species identification.

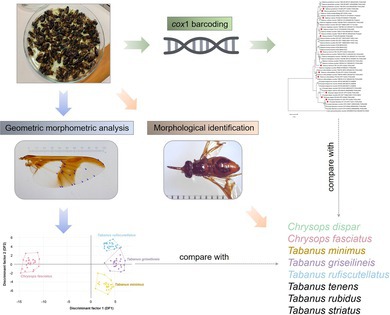

## INTRODUCTION

Tabanidae, blood‐sucking insects, belonging to the order Diptera, are composed of 177 genera and about 4665 species (Evenhuis & Pape, 2024). Most of the economically important tabanids are found in Chrysopsinae, including *Chrysops* spp. (deer fly), and the Tabanidae, including *Tabanus* spp. (horse fly) (Mullens, 2002). Thailand is a tropical country where various tabanid species are abundantly found and approximately 128 species of Tabanidae have been identified, including the genera *Tabanus* (86 species), *Haematopota* (36 species) and *Chrysops* (6 species) (Burton, 1978; Changbunjong et al., 2020; Stone & Philip, 1974). *Chrysops dispar* has been reported in Chumphon, Kanchanaburi, Lampang, Nakhon Prathom, Nakhon Ratchasima, Nakhon Si Thammarat and Sa Kaeo Provinces of Thailand (Changbunjong et al., 2020; Sontigun et al., 2022). *C. fasciatus* was found in Chon Buri, Kanchanaburi and Nakhon Ratchasima provinces (Changbunjong et al., 2020). In terms of *Tabanus* distribution, *T. griseilineis* and *T. rufiscutellatus* have been reported in Nakhon Ratchasima Province of Thailand, while *T. minimus* has been found in Chon Buri, Chumphon and Phra Nakhon Si Ayutthaya Provinces (Changbunjong, Sedwisi, et al., 2018).

Female tabanids served as vectors of several human and animal pathogens and gained high medical and veterinary importance (Sontigun et al., 2022). Tabanid flies have also been reported to attack humans, particularly in areas with few animals (Mullens, 2019). Therefore, controlling tabanid flies is necessary to break the chain of infection and its consequence (Mackerras et al., 2008). The correct identification of insect species is important for developing effective control programmes and management strategies (Ardkhongharn et al., 2023).

Morphological‐based identification of tabanid species is based mainly on the structures of the head, the characters of the callus, antennae, eyes, frons, beard, the colour and patterns of the insect body, wings and legs (Alikhan et al., 2018). This method is relatively economical since it does not require any complicated equipment but requires experienced taxonomists who are in short supply (de Lima et al., 2024). Using this technique, obtaining a complete structural sample is essential for accurate species identification. Additionally, because adult tabanids have complex structures and exhibit high diversity (while the morphological characteristics of some species are nearly identical), this conventional method is both labor‐intensive and time‐consuming.

In Thailand, DNA barcoding studies have been conducted for the species identification of *Tabanus* (horse fly) collected from 15 provinces of Thailand, including Chon Buri (East); Prachuap Khiri Khan, Chumphon, Songkhla (south); Kanchanaburi (West); Phra Nakhon Si Ayutthaya, Nakhon Prathom, Chainat, Singburi, Uthai Thani (central); Phitsanulok, Loei, Chiang Mai and Mae Hong Son (north); Nakhon Ratchasima (northern east) (Changbunjong et al., 2020). To date, no surveys have been conducted in the provinces of Narathiwat (southernmost) or Phayao (Northernmost).

Similar to DNA barcoding, a landmark‐based geometric morphometric (GM) analysis of insects has proven to be a promising alternative approach for not only the identification of genus and species, but also geographic and sex discrimination of a species (Oliveira‐Christe et al., 2020; Sauer et al., 2020). GM analysis is a method that can also be used to study the variation in size and shape within the same species. It can be performed using various methods, such as landmark, semi‐landmark and outline‐based methods, depending on the characteristics and specifics of insects (Dujardin, 2008; Dujardin et al., 2014; Dujardin & Dujardin, 2019). GM for species identification has been performed in various insects, including blow flies, flesh flies, mosquitoes, *Stomoxys* flies, sand flies and tsetse flies (Changbunjong et al., 2021; Giordani et al., 2017; Godoy et al., 2018; Kaba et al., 2017; Martinet et al., 2021; Sontigun et al., 2017; Sontigun et al., 2019). To date, in Thailand, although some landmark‐based geometric morphometrics have been performed (Changbunjong et al., 2021), relatively few studies have been conducted on wing morphometric analyses of Tabanidae flies.

Since more information on tabanid fly species of collected from various sites will provide more information on the species distribution and genetic diversity among Thai tabanid fly species. The present study aims to update a species list for Thailand and to compare and contrast identification methods involving keys based on classical morphology, geometry of wing venation and DNA barcoding using a single gene.

## MATERIALS AND METHODS

### Animal ethics

This study was reviewed and approved by the Siriraj Animal Care and Use Committee (SiACUC), Faculty of Medicine Siriraj Hospital, Mahidol University, Thailand (project code, SI‐ACUP 011/2566; COA No. 014/2566).

### Study sites

**FIGURE 1:**
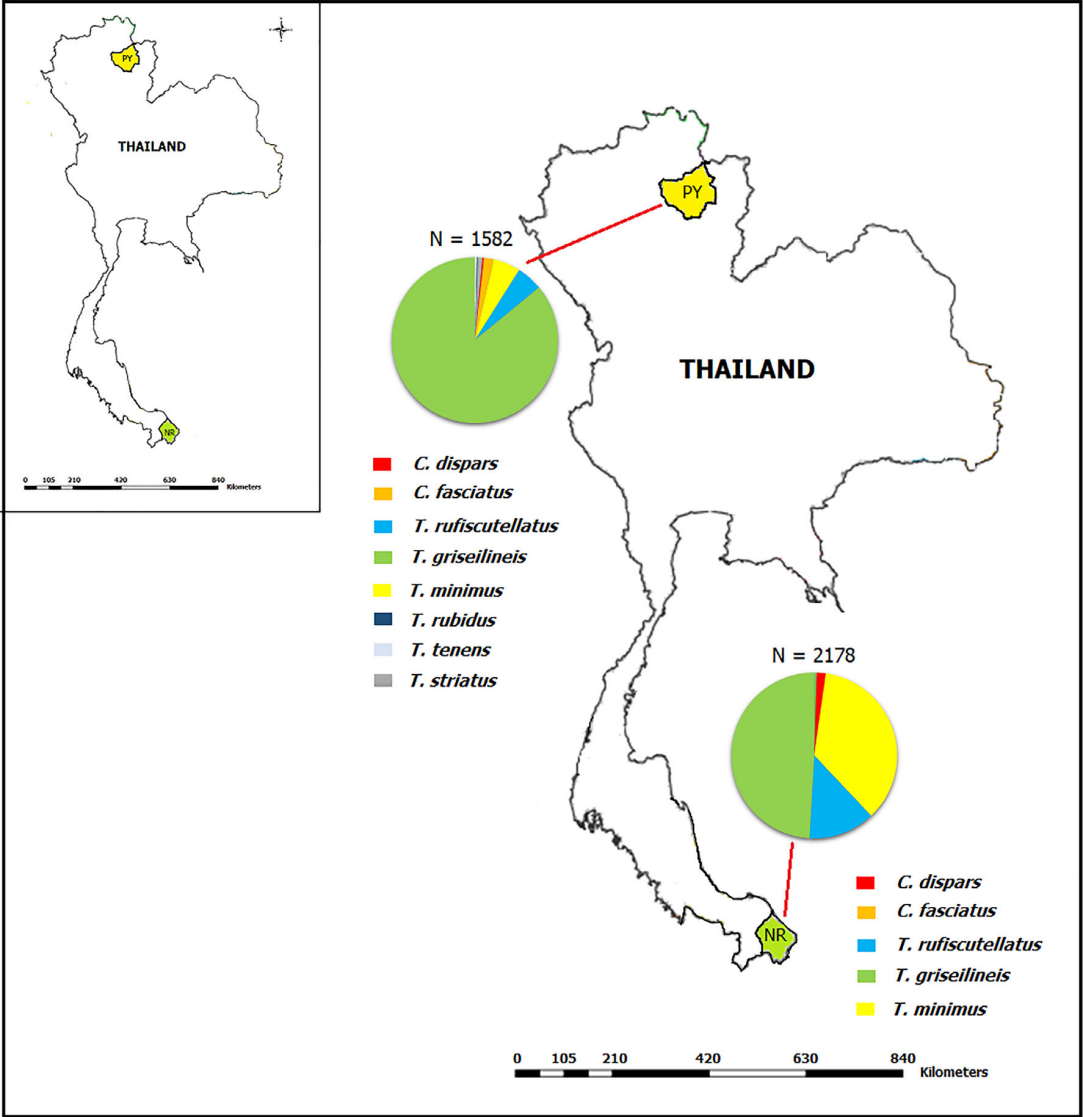
Collection sites of tabanid flies. Map showing the locations of Narathiwat (NR) and Phayao (PY) provinces of Thailand where the tabanid flies were collected. The species composition and number of tabanid flies collected at each site are illustrated in the accompanying pie charts.

Samples used in the present study are archived tabanid samples. Study sites were two farms located in the same area of Phayao Province (Mueang District; 19°9′57.251″N, 99°54′5.114″ E), northern Thailand, and three farms located in Narathiwat Province, including Mueang District (6°25′35.08″N, 101°49′19.689″ E), Ra‐ngae District (6°14′37.083″N, 101°41′38.026″ E) and Su‐ngai Padi District (6°5′8.822″N, 101°52′44.468″), southern Thailand (Figure 1). Each farm contained approximately 10 cattle and no other animal hosts. The country has three main seasons: winter, raining and summer. In the northern region, weather is less humid compared to the south due to the effect of the monsoon in the latter, while the winter season is drier, colder and longer, and the rainy season is less humid. The northern region is landlocked, while the south is bordered by seas to the east and west. The tabanid flies were caught feeding on roaming cattle.

### Study design and sample collection

Archived 1582 and 2178 female tabanid flies were collected from the provinces of Phayao and Narathiwat, Thailand, respectively, between October 2014 and March 2015, and preserved in 70% ethanol (v/v) at room temperature. They were obtained from two farms in Phayao Province and three farms in Narathiwat Province, using a hand collecting technique with 5‐ml collection vials (16 × 56 mm). Collections were conducted every other day during the daytime by catching flies perched on cattle, with approximately 8–10 specimens collected per day. Each sample was individually preserved in 70% ethanol (v/v) at room temperature and transferred to the laboratory at the Department of Parasitology, Faculty of Medicine Siriraj Hospital, Mahidol University, Bangkok, Thailand, with secure access. The ethanol solution was checked monthly to ensure that it had not evaporated.

### Morphological identification of tabanid fly using dichotomous keys

Tabanid samples (3760 female flies) were identified at the species level according to gross characters using the standard dichotomous taxonomic identification keys developed by Cornelius B. Philip (1960) and other available taxonomic literature (Burton, 1978; Philip, 1960) under a stereomicroscope (Nikon SMZ745; Nikon Corp., Tokyo, Japan).

### Molecular identification of tabanid flies using DNA barcoding

Half of the insect's body was extracted for genomic DNA using the QIAamp DNA Mini Kit (QIAGEN GmbH, Hilden, Germany) according to the manufacturer's protocol and stored at −20°C until use. DNA concentration was measured using a spectrophotometer (Nanodrop 1000, Thermo Scientific, Waltham, MA, USA). PCR was carried out using universal primers for mitochondrial cytochrome *c* oxidase subunit I (*cox*1) forward primer‐ LCO 1490 (5′‐GGTCAACAAATCATAAAGATATTGG‐3′) and reverse primer‐ HCO 2198 (5′‐TAAACTTCAGGGTGACCAAAAAATCA‐3′) (Folmer et al., 1994).

PCR assays were performed in a 25 μL per reaction, which contain 50 ng of genomic DNA, 500 nM of each primer and 1x AccuStart™ II GelTrack PCR SuperMix (Quantabio, Massachusetts, USA). DNA from the reference species of tabanid, as well as distilled water, was included in each experiment as positive and negative controls. The cycling conditions consisted of an initial denaturation step of 94°C for 5 min, followed by 35 cycles of 94°C for 30 s, 44°C for 1 min and 72°C for 1 min, and a final extension at 72°C for 5 min. Amplicons were separated by 1.5% agarose gel electrophoresis, stained with Green Stain Fluorescent Nucleic Acid Stain (Cyanagen, Bologna, Italy), and visualized under UV illumination (Bio‐Rad Laboratories, Hercules, CA, USA). Amplicons were sent for nucleotide sequencing. Sequence alignment and sequence analysis were performed with ClustalW (Thompson et al., 1994). The forward and reverse sequences of the *cox*1 gene were analysed and edited using BioEdit Sequence Alignment Editor software version 7.2.5 (Hall, 1999) and deposited in GenBank (accession numbers PP112238‐PP112247 and PV426456‐PV426458). For species identification, the consensus sequences were analysed in terms of sequence similarity to publicly available sequences in the NCBI GenBank database using the Basic Local Alignment Search Tool for Nucleotide (BLASTN) (Altschul et al., 1997) (https://blast.ncbi.nlm.nih.gov/Blast.cgi) and the Barcode of Life Data Systems (BOLD) using the Barcode Identification Engine (https://id.boldsystems.org/). Phylogenetic analysis was analysed based on maximum‐likelihood (ML) analysis with Kimura 2‐parameter (K2P) method (Kimura, 1980) and 1000 bootstrap replications using Molecular Evolutionary Genetics Analysis (MEGA) software version 10.0 (Kumar et al., 2018). The *cox*1 nucleotide sequence of *Culex quinquefasciatus* (MK265734) from the GenBank database was used as an outgroup. Moreover, we also generated the phylogenetic tree for the *Tabanus striatus* complex in Thailand, using the *cox*1 nucleotide sequence of *Chrysops fasciatus* (PP112240) as an outgroup. All tabanid samples used for DNA barcoding were the same individuals identified by key.

### Wing geometric morphometric (WGM) analysis

#### Study sample for WGM analysis

Study samples were chosen according to the requirement that there should be at least 40 samples for each species (at least twice the number of landmarks used). The right wing of each female sample was prepared on a permanent slide and photographed as JPG file with 150 dpi resolution under a stereomicroscope (Nikon SMZ745; Nikon Corp., Tokyo, Japan) connected to a WiFi Microscope Digital Camera Model MC4KW‐G1 (Microscope X, JiangSu, China). However, only 25 specimens per species were analysed in this study. This number was chosen to maintain equal sample sizes across species for comparison and to ensure that only specimens with complete and usable wing images were included. All samples used for WGM were the same individuals that had been morphologically identified using taxonomic keys. Pseudoreplication was not applied in this study.

#### Software

The XYOM (XY Online Morphometrics) online application software version 3 was used to digitize landmarks, perform statistical analysis and generate graphical output (Dujardin & Dujardin, 2019). This morphometric software is freely accessible at https://xyom.io.

#### Landmark digitization

**FIGURE 2:**
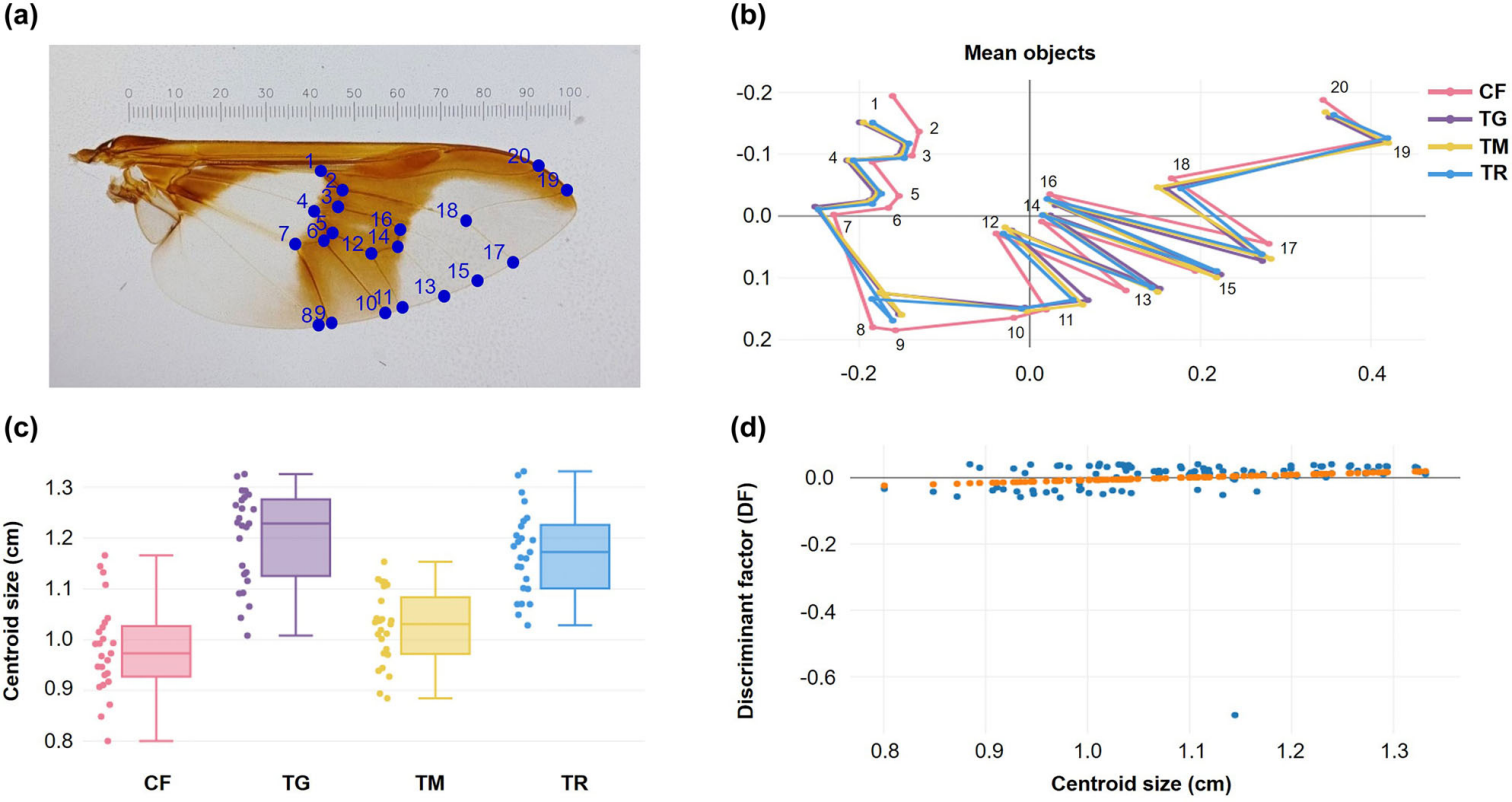
Wing geometric morphometric analysis; **(a)** Position of the 20 landmarks (LM), **(b)** Mean anatomical landmark configurations of individual wing vein after Procrustes superposition, **(c)** Boxplots illustrating wing centroid size variation among four *Tabanus* species. The horizontal line within each box represents the median, with the 25th and 75th quartiles shown. Coloured dots represent individual samples, **(d)** Linear regression analysis of the discriminant factor on the centroid size of tabanid flies. The blue dots represent the individual discriminant and centroid size values, while orange dots represent the linear regression prediction; CF, *C. fasciatus*, TG, *T. griseilineis*, TM, *T. minimus* and Tr, *T. rufiscutellatus*.

The right wing of each female tabanid was detached from the thorax using a sterile blade, placed on a glass microscope slide, dropped by a drop of Hoyer's medium, covered with a glass cover slip and left to dry at room temperature for a week. Wing pictures were photographed in dorsal view (with the costal and apical margins positioned upward and to the right, respectively) with a stereomicroscope with a scale bar at 40x magnification (Ruenchit & Wongkamchai, 2025) and uploaded to the XYOM online application software. Twenty landmarks on each wing image were digitized in specific order (Figure 2a) (Table 1). These landmarks were defined following the study by Altunsoy et al. (2017) (Altunsoy et al., 2017). The scale bar was set to prevent error in sizing. The raw coordinates of the landmarks were recorded for further characterization and classification analyses. In this study, 25 samples of each *Tabanus* species were randomly selected for GM analysis, based on the completeness of their wing images. Additionally, 25 samples of *Chrysops fasciatus* were included to generate in‐house reference data for further species identification of unknown specimens.

**TABLE 1:** Landmark scheme and venation terminology.

Landmark No.	Venation terms	Landmark No.	Venation terms
L1	Intersection of veins R_s_ and R_2+3_	L11	Basal end of vein M_3_
L2	Intersection of veins R_4_ and r‐m	L12	Intersection of veins M_3_ and m‐m
L3	Intersection of veins M1 and r‐m	L13	Basal end of vein M_2_
L4	Intersection of veins br and d	L14	Basal of vein M_2_
L5	Intersection of veins m‐cu and M_3_	L15	Basal end of vein M_1_
L6	Intersection of veins m‐cu and M_4_	L16	Intersection of veins m‐m and M_2_
L7	Intersection of veins m‐cu and CuA	L17	Basal end of vein R_5_
L8	Intersection of veins A_1_ and CuA	L18	Intersection of veins R_4_ and R_5_
L9	Basal end of vein CuA	L19	Basal end of vein R_4_
L10	Basal end of vein M_4_	L20	Basal end of vein R_2+3_

#### Repeatability test

Thirteen wing samples per species (52 images in total) were randomly selected and digitized twice by the same operator to estimate the precision of the landmark digitization. Specimen labels were blinded during digitization. The repeatability of the shape was evaluated by Procrustes analysis using the two sets of raw coordinates of the landmarks as input variables. Analysis was performed using XYOM software. The result was expressed as a repeatability score.

#### Characterization and classification of wing shape and wing size

Wing shape variation was observed by visual comparison of superposed configurations using Generalized Procrustes Analysis in XYOM software. The result was shown as the mean shape of each group of samples. The centroid size was analysed on the basis of the distances between the centroid point and each landmark to estimate the variation of the wing size between the groups of tabanid flies. The result was represented by the size distribution histogram. One‐way analysis of variance (ANOVA) was used to compare the mean centroid size among different tabanid groups. A nonparametric permutation test (1000 permutations) with Bonferroni correction was performed to determine the statistically significant difference of the one‐way ANOVA with a value of *p* of 0.05. The factor map of the first two discriminant factors (DFs) was also analysed by discriminant analysis (DA) using the final wing shape variables (*n* = 21) to observe the separation between groups of tabanids. The Mahalanobis distances were calculated to estimate the metric distance between the groups of tabanids. The classification precision score of each species and the total performance score were recorded.

#### Allometry analysis

The allometric effect that highlights a contribution of size to shape variation was analysed using linear regression of the DF (shape variable) on the centroid size (size variable). The linear determination coefficient (*r* ^2^) was recorded.

### Identification of test samples

To evaluate the efficacy of our in‐house reference materials (the raw coordinates of the wing landmarks of the 100 female tabanid samples collected from Narathiwat province), the mounted right wings of the 4 species of tabanid, including *C. fasciatus* (unknown group 1, 5 samples), *Tabanus griseilineis* (unknown group 2, 25 samples), *T. minimus* (unknown group 3, 25 samples) and *T. rufiscutellatus* (unknown group 4, 25 samples), were used as test samples to identify using the following four reference groups in our study: (1) *C. fasciatus*, (2) *Tabanus griseilineis*, (3) *T. minimus* and (4) *T. rufiscutellatus*. The identification method was performed individually for the test samples. To classify test samples as members of a specific species, principal component analysis (PCA) was performed in the same manner as was performed for wing shape analysis by XYOM software, where the morphospace of the reference data was used for each test sample. In addition, the hierarchical agglomerative clustering (HAC) tree based on the Euclidean distances between the shape variables was analysed using the in‐house reference data. The similarity between test samples and the in‐house reference data was observed (Ardkhongharn et al., 2023).

## RESULTS

### Number of samples and areas of tabanid fly collection

A total of 3760 female tabanid flies were collected from Narathiwat (1582 females) and Phayao (2178 females) and species identification was performed using taxonomic keys, DNA barcoding and WGM analysis. Collection sites, as well as the composition of the tabanid fly species and number of samples, are shown in Figure 1 and Table 2.

**TABLE 2:** Identification of tabanid flies from Narathiwat and Phayao provinces, Thailand, based on dichotomous key and DNA barcoding analysis.

Province	Number caught samples	Identification method	
		Dichotomous key	DNA barcoding
Narathiwat	11	*Chrysops dispar*	*Chrysops dispar*
	38	*Chrysops fasciatus*	*Chrysops fasciatus*
	1069	*Tabanus griseilineis*	*Tabanus griseilineis*
	778	*Tabanus minimus*	*Tabanus minimus*
	282	*Tabanus rufiscutellatus*	*Tabanus rufiscutellatus*
Phayao	30	*Chrysops dispar*	NA
	5	*Chrysops fasciatus*	NA
	1360	*Tabanus griseilineis*	NA
	84	*Tabanus minimus*	NA
	80	*Tabanus rufiscutellatus*	NA
	6	*Tabanus* sp.	*Tabanus tenens*
	3	*Tabanus* sp.	*Tabanus rubidus*
	14	*Tabanus* sp.	*Tabanus striatus*

Abbreviation: NA, not applicable.

### Morphological identification using taxonomic keys

Among the 2178 female tabanid samples collected from Narathiwat, identified based on morphology, 2 species of *Chrysops* (11 samples of *Chrysops dispar* and 38 samples of *C. fasciatus*), along with 3 species of *Tabanus* (778 samples of *T. minimus*, 282 samples of *T. rufiscutellatus* and 1069 samples of *T. griseilineis*) were identified. For the 1582 female tabanid samples collected from Phayao, 2 species of *Chrysops* (5 samples of *C. fasciatus* and 30 samples of *C. dispar*), as well as 3 species of *Tabanus* (1360 samples of *T*. *griseilineis*, 80 samples of *T. rufiscutellatus* and 84 samples of *T. minimus*) were identified, along with unidentified samples to the species level of 23 samples (6, 3 and 14 samples of pool 1, 2 and 3, respectively) of genera *Tabanus*.

### DNA barcode analysis

Thirteen *cox*1 sequences were obtained in this study. The mean nucleotide composition of *cox*1 included 38.35% thymine (T), 15.59% cytosine (C), 29.14% adenine (A) and 16.92% guanine (G). These *cox*1 sequences were characterized by a high AT content (67.49%).

Samples of *C. dispar, C. fasciatus*, *T. minimus*, *T. rufiscutellatus* and *T. griseilineis*, each identified morphologically at the species level, were randomly selected for DNA barcoding. Additional 23 samples of *Tabanus* spp. that could only be classified to genus levels were also randomly subjected to DNA barcoding.

**FIGURE 3:**
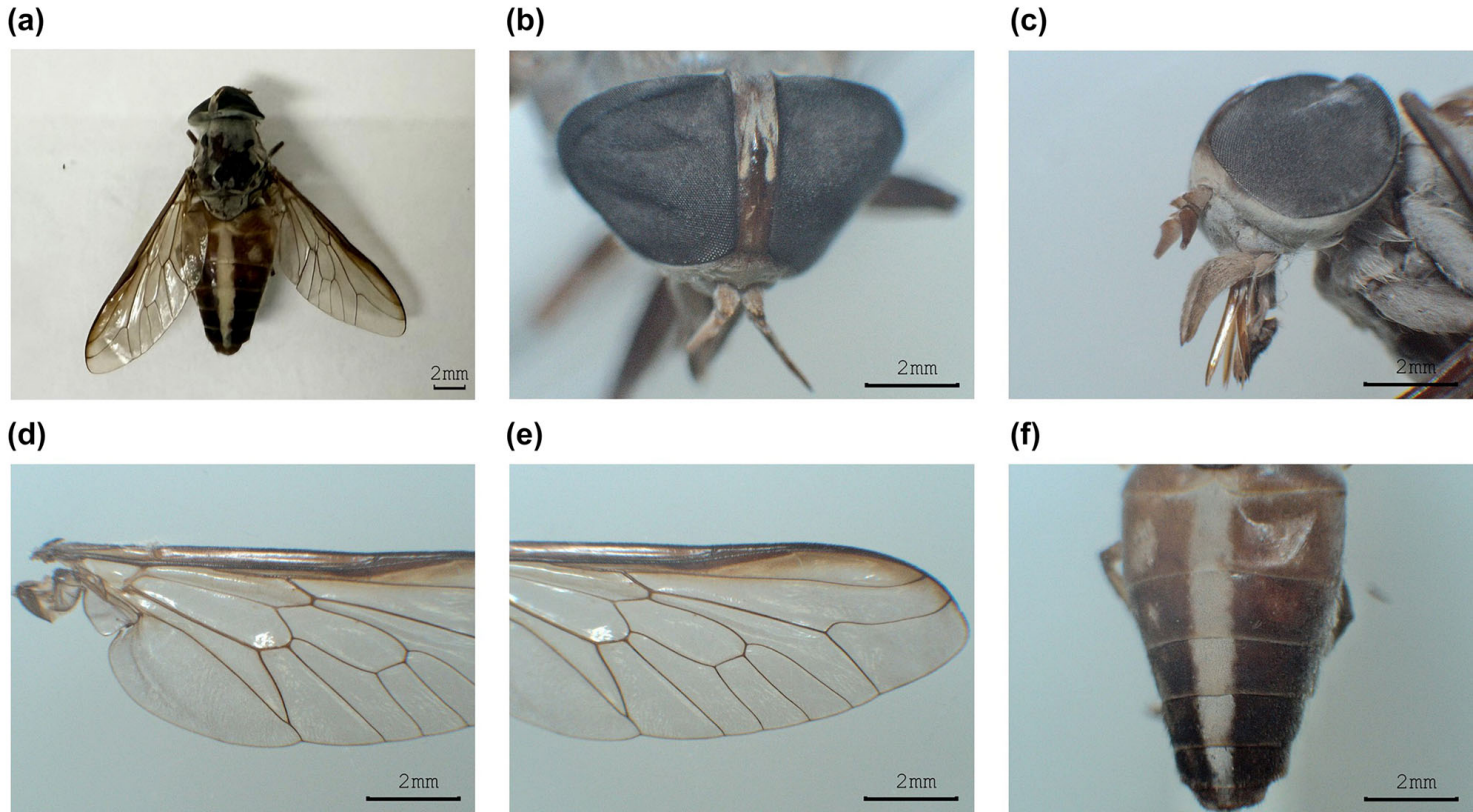
Images illustrating the key morphological characteristics of *T. tenens*. **(a)** Dorsal view of whole body. **(b)** Frontal view of head and antennae. **(c)** Lateral view of head and antennae. **(d–e)** Dorsal view of wing. **(f)** Dorsal view of abdomen.

The percentage identity of *cox*1 gene sequences generated in this study, compared to those available in the GenBank and BOLD databases, is shown in Table 3. The sequences of *C*. *dispar, C. fasciatus*, *T. minimus*, *T. rufiscutellatus* and *T. griseilineis* showed high identity (>90%) with their conspecific sequences. Among the samples initially identified at the genus level as *Tabanus* spp. based on morphological traits, they were identified as *Tabanus tenens*, *T. rubidus* and *T. striatus*. Morphological characteristics of *T. tenens* were shown in Figure 3. The sequences of *T. minimus* exhibited 94.26%–100% identity with *T. minimus* in the GenBank and BOLD databases. However, these sequences also showed a high identity with *T. mesogaeus* (94.72%–94.88%) and *T. formosiensis* (95.6%). Similarly, the *T. striatus* sequence demonstrated high identity with both *T. striatus* (96.02%–99.81%) and *T. megalops* (99.42%).

**TABLE 3:** Percentage identity of *Tabanus* spp. and *Chrysops* spp. cytochrome *c* oxidase subunit 1 (*cox1*) sequences retrieved in present study, compared with sequences previously deposited in the GenBank and BOLD databases.

ID	Species	Accession number	GenBank			BOLD		
			Species match	% identity	No. of analysed sequences	Species match	% identity	No. of analysed sequences
1	*C. dispar*	PP112238	*C. dispar*	97.10–99.35	37	*C. dispar*	97.92–98.75	23
2	*C. dispar*	PP112239	*C. dispar*	91.64–96.77	75	*C. dispar*	95.70–97.02	25
3	*C. fasciatus*	PP112240	*C. fasciatus*	98.29–99.14	10	*Chrysops* spp.	98.42–100	5
4	*C. fasciatus*	PP112241	*C. fasciatus*	98.66–99.16	10	*Chrysops* spp.	98.66–100	5
5	*T. rufiscutellatus*	PP112242	*T. rufiscutellatus*	99.78–100	3	*T. rufiscutellatus*	99.49–100	4
6	*T. rufiscutellatus*	PP112243	*T. rufiscutellatus*	98.93–99.20	3	*T. rufiscutellatus*	98.69–99.02	4
7	*T. minimus*	PP112244	*T. minimus*	94.00–98.33	15	*T. minimus*	95.19–100	10
			*T. mesogaeus*	95.10–95.74	3	*T. mesogaeus*	95.56–95.75	2
			*T. formosiensis*	95.59	1			
8	*T. minimus*	PP112245	*T. minimus*	94.33–97.67	13	*T. minimus*	94.78–100	8
			*T. mesogaeus*	94.72–94.88	3	*T. mesogaeus*	94.78–94.96	3
			*T. formosiensis*	95.59	1			
9	*T. griseilineis*	PP112246	*T. griseilineis*	95.56–96.50	3	*T. griseilineis*	96.37–98.60	8
10	*T. griseilineis*	PP112247	*T. griseilineis*	96.68–97.34	2	*T. griseilineis*	96.99–100	8
11	*T. tenens*	PV426456	*T. tenens*	97.34–99.29	4	*T. tenens*	98.17–100	5
12	*T. rubidus*	PV426457	*T. rubidus*	95.80–98.70	89	*T. rubidus*	99.04–99.36	23
13	*T. striatus*	PV426458	*T. striatus*	96.02–99.81	25	*T. striatus*	98.89–100	11
			*T. megalops*	96.15–99.42	15	*T. megalops*	99.33	4

### Phylogenetic analyses

**FIGURE 4:**
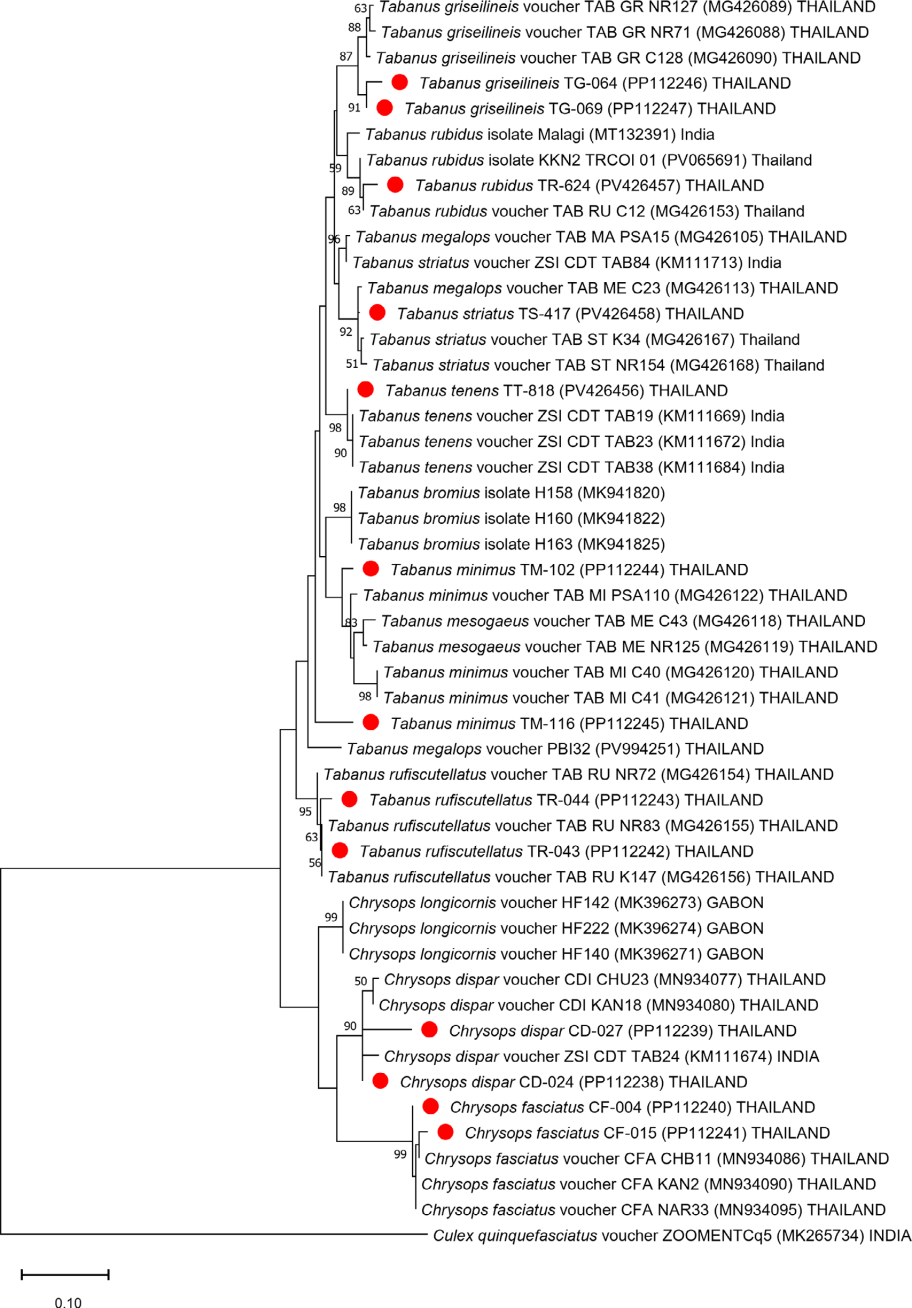
Maximum‐likelihood (ML) phylogenetic tree showing relationship between the newly identified *Chrysops* and *Tabanus* spp. and described species from the GenBank database based on nucleotide sequences of the *cox*1 gene. Numbers at nodes represent bootstrap values based on 1000 replications. Only groups with more than 50% bootstrap support are shown on the nodes. Bars represent average nucleotide substitutions per sequence position. The scale bar shows the number of substitutions per site. Red circles denote sequences from the present study; unmarked branches represent reference sequences.

**FIGURE 5:**
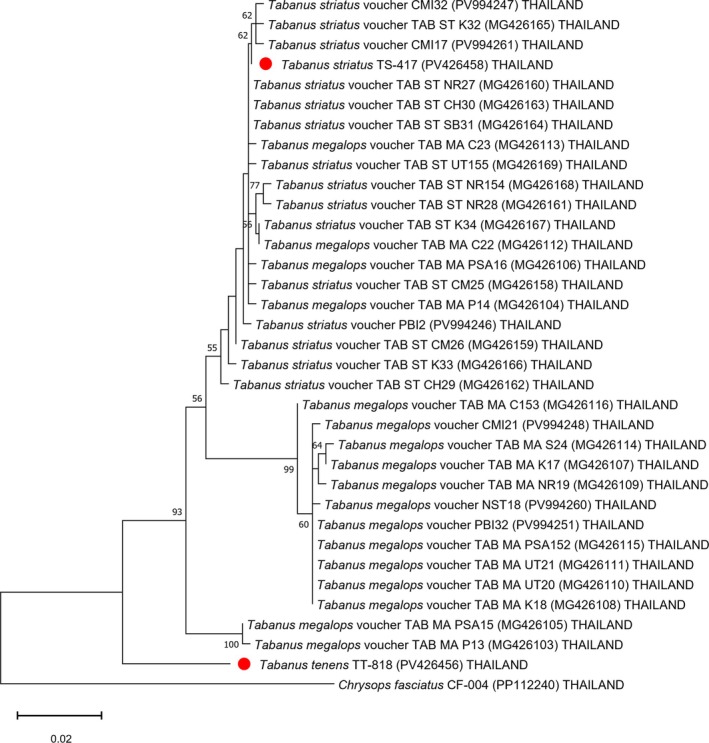
Phylogenetic tree analysis of the *cox*1 nucleotide sequences for the *Tabanus striatus* complex recorded in Thailand. *cox*1 nucleotide sequence of *Chrysops fasciatus* (PP112240) was used as an outgroup. Numbers at nodes represent bootstrap values based on 1000 replications. Only groups with more than 50% bootstrap support are shown on the nodes. Bars represent average nucleotide substitutions per sequence position. The scale bar shows the number of substitutions per site. Red circles denote sequences from the present study; unmarked branches represent reference sequences.

Maximum‐likelihood (ML) analysis based on *cox*1 gene sequences was generated using MEGA software with 1000 bootstrap replicates. The analysis included 49 sequences analysed: 13 sequences obtained from this study, 35 reference sequences retrieved from the GenBank database, and a sequence of *Culex quinquefasciatus* (MK265734) as an outgroup. As illustrated in Figure 4, the sequences of *C. dispar* (CD‐024 and CD‐027), *C. fasciatus* (CF‐004 and CF‐015), *T. rufiscutellatus* (TR‐043 and TR‐044), *T. griseilineis* (TG‐064 and TG‐069), *T. minimus* (TM‐102 and TM‐116), *T. tenens* (TT‐818), *T. rubidus* (TR‐624) and *T. striatus* (TS‐417) clustered with the respective reference sequences for these species. However, the sequences of *T. minimus* were grouped with the sequence of *T. mesogaeus*. Similarly, the *T. striatus* sequences were found to cluster with the *T. megalops* sequence. This finding corresponds to the blasting sequence. In addition, comparing the *cox*1 gene sequence of *T. tenens* found in the present study to the other possibly synonymous species in the *T. striatus* complex including *T. striatus* as well as *T. megalops* that have been reported in Thailand confirmed a new record of *T. tenens* in Thailand (Figure 5).

### Wing geometric morphometric analysis (WGM)

As shown in Figure 2a, WGM of the wing veins started with positioning of the 20 landmarks (LM) on the right wing of each tabanid sample by XYOM online application software at https://xyom.io. The scale bar was included in all images to set the sizing error. Before characterization and classification of the tabanid wing pictures, the precision of landmark digitization was estimated, and it was found that the shape repeatability score was high at 98.6% by Procrustes analysis. For wing shape variation, the mean anatomical landmark configurations of *C. fasciatus*, *T. griseilineis*, *T. minimus* and *T. rufiscutellatus* after Procrustes superposition are shown in Figure 2b. Most visible landmark displacements were found at landmarks 1, 2, 4, 5, 6, 8, 11 and 13 (Figure 2b).

For wing size variation, the results demonstrated that centroid size variation was found among tabanid flies (Figure 2c). The wings of *T. griseilineis* are the largest (mean = 1.20 cm), followed by *T. rufiscutellatus* (mean = 1.17 cm), *T. minimus* (mean = 1.02 cm) and *C. fasciatus* (mean = 0.98 cm) (Table 4), respectively. *T. griseilineis* had a significantly larger wing size than *T. minimus* and *C. fasciatus* (*p* < 0.001), while *T. rufiscutellatus* had a significantly larger wing size than *T. minimus* and *C. fasciatus* (*p* < 0.001). This variation in wing size is consistent with the body size observed by morphological identification, *T. griseilineis* (1.95 cm), *T. rufiscutellatus* (1.4 cm), *T. minimus* (1.05 cm) and *C. fasciatus* (1.0 cm), indicating a relationship between body size and wing size.

**TABLE 4:** Variation in wingspan of each tabanid fly.

Tabanid species	*N*	Mean (min – Max) (cm)	Variance	SD
*Chrysops fasciatus* (CF)	25	0.98 (0.80–1.17)	0.00	0.09
*Tabanus rufiscutellatus* (TR)	25	1.17 (1.03–1.33)	0.00	0.08
*Tabanus minimus* (TM)	25	1.02 (0.88–1.15)	0.00	0.07
*Tabanus griseilineis* (TG)	25	1.20 (1.01–1.33)	0.00	0.09

**FIGURE 6:**
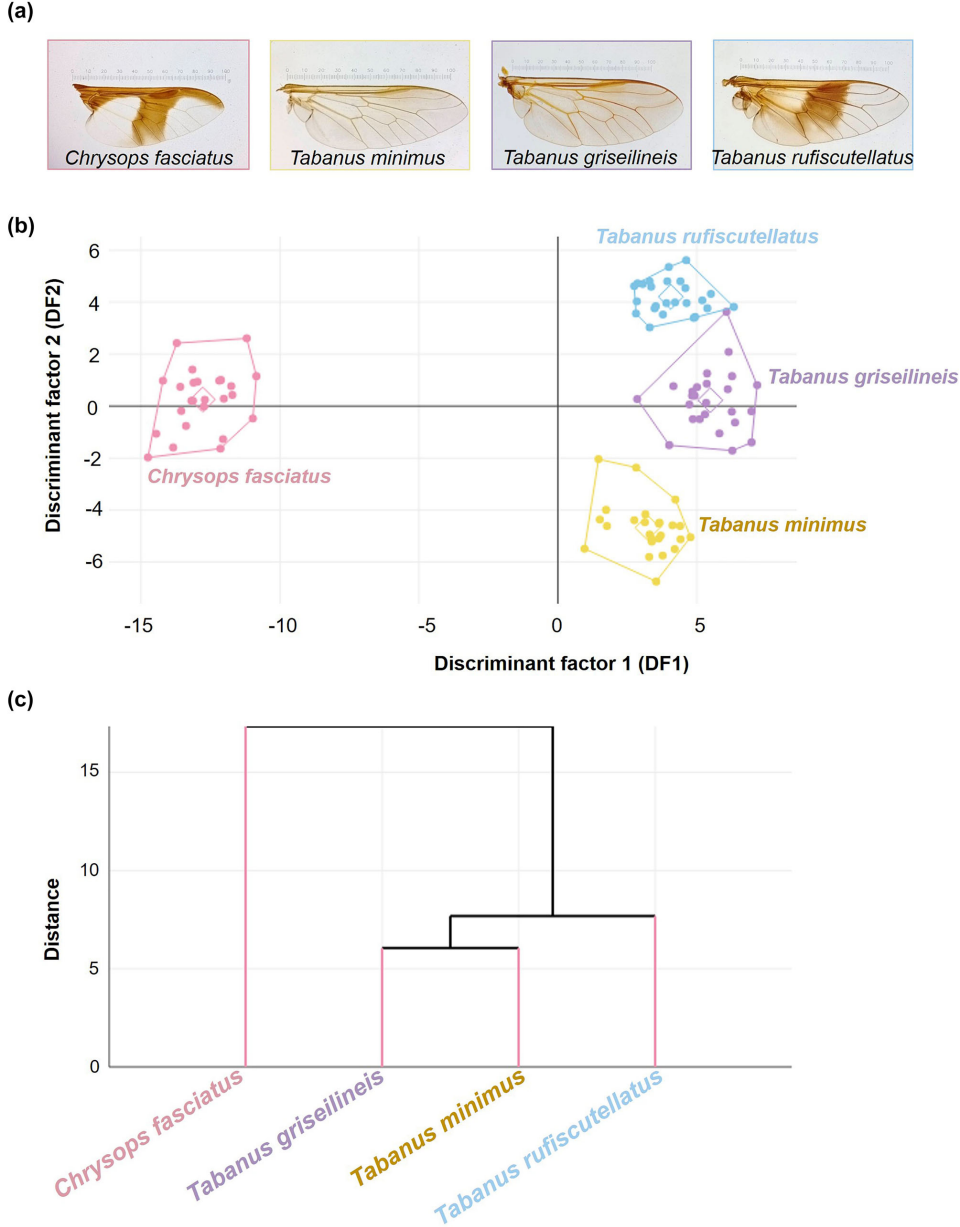
Discrimination analysis of tabanid fly samples using geometric morphometric analysis. **(A)** Wing pictures of each tabanid fly species. **(B)** Factor map of the two discriminant factors (DF1, 14.6% of the variance as horizontal axis and DF2, 4.3% of the variance as vertical axis) of *C. fasciatus*, *T. griseilineis*, *T. minimus* and *T. rufiscutellatus*. **(C)** Hierarchical clustering tree of tabanid flies analysed based on the Mahalanobis distances of principal components of the four tabanid species: *C. fasciatus*, *T. griseilineis*, *T. minimus* and *T. rufiscutellatus*.

Furthermore, to observe an allometric effect that demonstrates an effect of wing size on wing shape variation, the result showed that the discrimination power between four species based on wing shape was not influenced by wing size. The linear determination coefficient (*r* ^2^) is very low at 1.7% (Figure 2d). Additionally, to evaluate the potential of species discrimination using WGM analysis, the individual plot was observed in the two derived DFs shaped (DF1 and DF2). The results showed that a *Chrysops* plot was distinctly separated from those of three species of *Tabanus* (Figure 6b). Among *Tabanus*, the samples of *T. rufiscutellatus*, *T. griseilineis* and *T. minimus* were also discriminated (Figure 6b). Classification precisions were 100% for *C. fasciatus*, *T. minimus* and *T. rufiscutellatus*, while 96% for *T. griseilineis* (Table 5). The total performance score was 99% (Table 5). The hierarchical clustering tree based on Mahalanobis distances of the four tabanid species showed that the shape of the wing of *T. griseilineis* was more similar to *T. minimus* than *T. rufiscutellatus*, while *C. fasciatus* was separated (Figure 6c).

**TABLE 5:** Percentages of accuracy score for tabanid fly collected from Narathiwat based on wing shape using a cross‐validated classification.

Tabanid species	Accuracy (assigned/observed)
*Chrysops fasciatus* (CF)	100% (25/25)
*Tabanus rufiscutellatus* (TR)	100% (25/25)
*Tabanus minimus* (TM)	100% (25/25)
*Tabanus griseilineis* (TG)	96% (24/25)
Total performance	99% (99/100)

### Species identification of test samples

**FIGURE 7:**
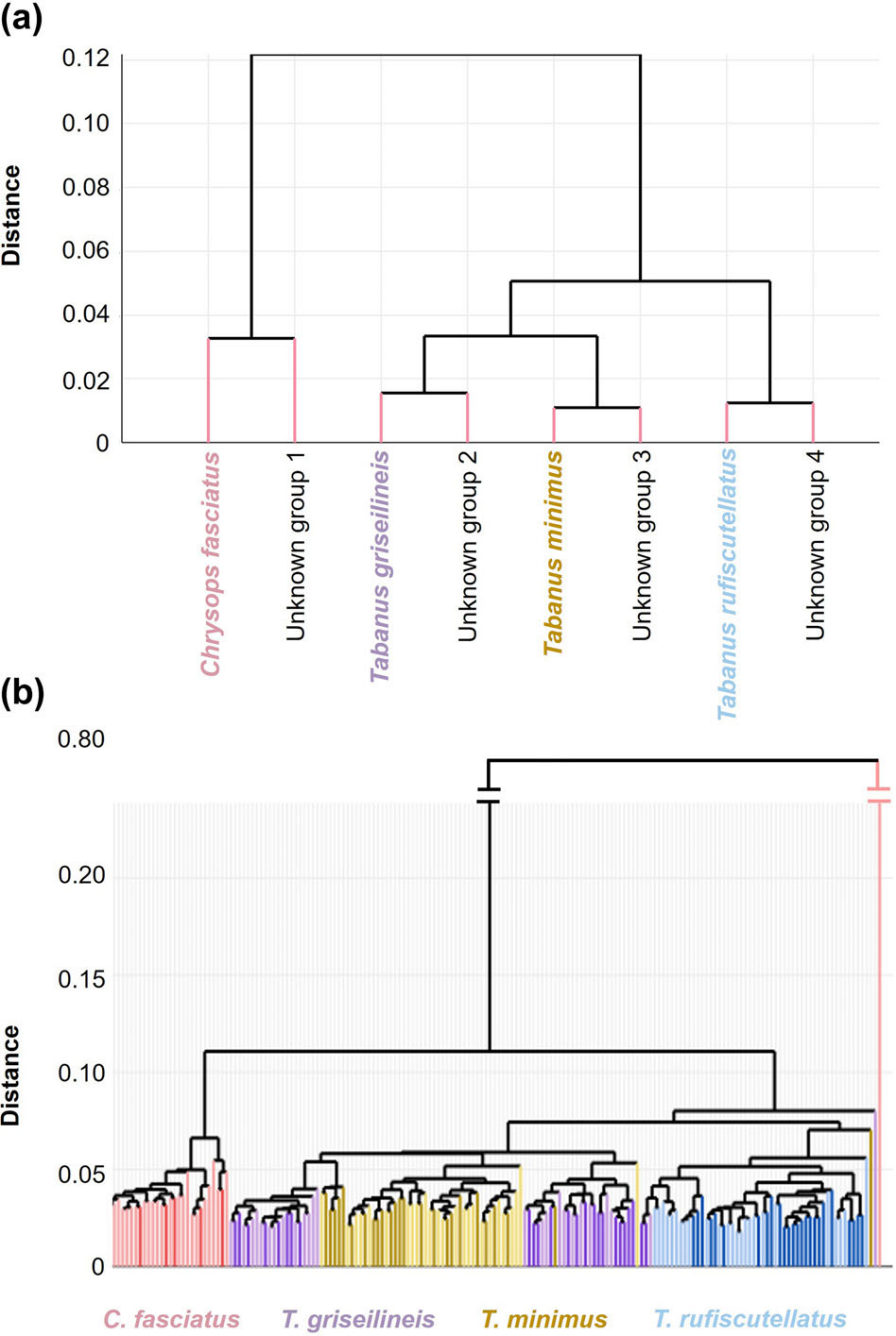
Species identification of tabanid flies collected from different geographical location using in‐house reference data generated from Narathiwat tabanid samples. **(a)** The hierarchical clustering tree based on the Euclidean distances between average group shapes in the morphospace. **(b)** Hierarchical agglomerative clustering (HAC) tree generated by an unsupervised method showing similarities between individual sample [dark red lines (5 samples of *C. fasciatus*), dark purple lines (25 samples of *T. griseilineis*), dark yellow lines (25 samples of *T. minimus*) and dark blue lines (25 samples) of *T. rufiscutellatus*, total = 80 samples) and reference groups.

To test the efficacy of WGM analysis for species identification of samples from different geographical areas, in‐house reference data gathered from the raw coordinates of wing landmarks of Narathiwat tabanid samples was used to identify species of tabanid flies collected from Phayao province (test samples, 80 samples). Based on the UPGMA algorithm, results showed that the unknown groups 1 to 4 were classified into the same groups with *C. fasciatus*, *T. griseilineis*, *T. minimus* and *T. rufiscutellatus*, respectively (Figure 7a). Based on the HAC tree, all test samples of unknown groups 1, 3 and 4 were correctly assigned to *C. fasciatus*, *T. minimus* and *T. rufiscutellatus*, respectively. However, some samples of unknown group 2 were correctly assigned to *T. griseilineis*, while some of them were incorrectly assigned (Figure 7b).

## DISCUSSION

In the present study, *C. dispar*, *C. fasciatus*, *T*. *griseilineis*, *T*. *minimus*, *T*. *rufiscutellatus* were detected in Narathiwat, the southernmost province, and *C. dispar*, *C. fasciatus*, *T*. *griseilineis*, *T*. *minimus*, *T*. *rufiscutellatus, T. tenens, T. rubidus* and *T. striatus* were detected in Phayao, the northernmost province of Thailand. To our knowledge, *T. tenens* is a new recorded species of Thai horse fly, one of the *Tabanus striatus* complex which includes *T*. *striatus*, *T. tenens* and *T. dorsiger*. These flies have been reported to be vectors of trypanosomiasis (Banerjee et al., 2015; Veer, 2004).

To date, relatively few studies have been conducted on the species diversity of tabanid flies in Thailand (Changbunjong et al., 2025; Changbunjong, Bhusri, et al., 2018; Tumrasvin, 1989). Therefore, our study will increase knowledge about the distribution of tabanid flies in Thailand. We detected more species of tabanid flies from Phayao compared to those collected from Narathiwat. This finding is consistent with a previous study in Thailand, which indicated that horse fly species were concentrated in the northern parts of Thailand (Tumrasvin, 1989). The northern Thailand has a tropical climate and geographically characterized by several mountain ranges, river valleys and forested environments, thus, creating suitable habitats for tabanid fly.

We used DNA barcoding to discriminate among horse flies that belong to five morphologically distinct species. The DNA barcoding also helped to identify three more species of tabanid fly identified only to genus level by morphological based, that is, *T. tenens*, *T. rubidus* and *T. striatus*. Furthermore, the three species within the *Tabanus striatus* complex, including *T*. *striatus*, *T. tenens* and *T. dorsiger*, have been misidentified and considerable data on different biological parameters have been developed under incorrect names (Burger & Thompson, 1981). These three species could be clearly distinguished based on DNA barcoding (Banerjee et al., 2015), revealing the ability of DNA barcoding as an alternative tool for the identification of the species of the tabanid fly. Changbunjong et al. indicated that the *cox*1 gene marker cannot reliably differentiate between *T. minimus* and *T. mesogaeus* and *T. striatus* and *T. megalops*, suggesting that additional molecular markers are required for precise identification (Changbunjong, Bhusri, et al., 2018). The result of our study is consistent with previous research. *Tabanus mesogaeus* can be separated from *T. minimus* on the basis of a combination of morphological characteristics, including blackened beard, absence of pale hair on the thorax and abdomen and facial brown tomentum (Burton, 1978; Changbunjong, Bhusri, et al., 2018). However, distinguishing these morphological features requires a lot of taxonomy‐based experience and is extremely time consuming, which may lead to misidentifications.

In Thailand, few molecular studies have been conducted on the tabanid fly with respect to its identification of molecular species. The coverage of tabanid fly species in the reference database is a critical factor for accurate species identification using the DNA barcoding technique. Thus, the data obtained from the present study will increase the barcode databases of the NCBI GenBank and expand the species coverage of the actual list of tabanid distribution.

For WGM, the use of a cross‐validation test demonstrated that the shape of the wings can be used to evaluate species levels with high reliability. The wing shape of *T. minimus* shows a little overlap with that of *T. griseilineis*, but the cross‐validation revealed a relatively high percentage (88%–100%) of correct classification in all studied species. Our study highlighted that WGM can classify tabanid species with an overall accuracy of 99%, even when only 20 landmarks were used. For the analysis of the variation of wing size, a variation of centroid size was found between species of tabanid flies, but this variation in wing size is consistent with the body size observed by morphological identification, indicating a relationship between body size and wing size.

The present study also demonstrated the use of WGM data from tabanid species collected from Narathiwat province in southern Thailand as in‐house reference material to identify new field samples (test samples) collected from Phayao province, in northern Thailand. Based on the UPGMA algorithm, results showed that overall test samples could be differentiated into the same groups as *C. fasciatus*, *T. rufiscutellatus*, *T. minimus* and *T. griseilineis*, respectively. However, for species‐specific identification using the HAC tree, which focuses on individual samples, all three test sample groups were correctly assigned to *C. fasciatus*, *T. minimus* and *T. rufiscutellatus*, whereas some samples of *T. griseilineis* were incorrectly assigned. This indicates that landmark‐based identification has a low discrimination power for *T. griseilineis* identification, suggesting a limitation of this method.

WGM analysis offers a cost‐effective and accessible method for studying morphological traits and differentiating insect species for people who have limited knowledge in taxonomy. WGM is a statistical analysis of patterns of change in the shape of the anatomical structure; the minimum number of samples per species is required for precise differentiation. The mean centroid size is highly accurate, even with small sample sizes, since errors are typically much smaller than the differences observed between populations. In contrast, the estimation of the mean shape requires larger sample sizes. Overall, both centroid size and shape variance need at least 15–20 samples to obtain an acceptable level of accuracy (Cardini et al., 2015). In the present study, the accuracy of the WGM technique for identifying tabanid flies is proportional to the available data, which are still limited. Two species of *Chrysops* collected from Narathiwat and Phayao could not be recruited to perform the WGM analysis due to the small number of samples collected. This is a limitation of WGM analysis. Another limitation of WGM is the requirement for the samples to be in good physical condition, especially for delicate structures such as insect wings that can easily be damaged. The final limitation is the lack of widely available reference databases, which makes species identification challenging. Therefore, expanding morphometric reference databases and making them publicly available will enhance the accuracy and efficiency of these tools. By sharing these resources beyond internal use, we can encourage collaboration and ensure that the data are accessible to researchers worldwide, promoting more comprehensive and reliable analyses. These would provide better insight into the diversity of the tabanid fly and its potential role in disease transmission.

Overall, although the three species identification methods are now widely recognized as standard approaches, the findings of the present study underscore their respective strengths and limitations. Notably, these methods constitute essential tools for updating the species inventory of tabanid flies in Thailand.

## CONCLUSIONS

We provided DNA barcode data of eight species of tabanid flies along with a new record of the Thai horse fly, *T. tenens*. WGM can also differentiate five species of tabanid, and our WGM data obtained from WGM of tabanid collected from Narathiwat could be successfully used as an in‐house reference material to differentiate tabanid collected from other locations in Thailand. Importantly, this study displayed a regional tabanid diversity using an integrative identification toolkit.

## AUTHOR CONTRIBUTIONS

**Nantatchaporn Klaiklueng:** Data curation; formal analysis; investigation; visualization; writing – original draft. **Weluga Bootsongkorn:** Investigation; methodology; resources. **Patsharaporn T. Sarasombath:** Data curation; investigation. **Pichet Ruenchit:** Conceptualization; formal analysis; methodology; funding acquisition; writing – original draft; writing – review and editing. **Sirichit Wongkamchai:** Conceptualization; supervision; resources; writing – review and editing.

## FUNDING INFORMATION

This work was supported by Siriraj research development fund, Grant number (IO) R016637003, and Siriraj Core Research Facility (SICRF), Faculty of Medicine Siriraj Hospital, Mahidol University, Thailand.

## CONFLICT OF INTEREST STATEMENT

The authors declare no conflicts of interest.

## ETHICS STATEMENT

The Animal Ethics Committee of the Faculty of Medicine Siriraj Hospital at Mahidol University, operating under the National Research Council of Thailand, approved the animal experiment of this study (project code, SI‐ACUP 011/2566; COA number 014/2566).

## Data Availability

All data generated or analysed during this study are included in this published article. The newly generated sequences were submitted to the GenBank database (https://www.ncbi.nlm.nih.gov/) under the accession numbers PP112238‐PP112247 and PV426456‐PV426458. Wing images used for geometric morphometric analysis were deposited in the Dryad repository https://doi.org/10.5061/dryad.kh18932nm.
